# The Effect of Ultraviolet Photofunctionalization on a Titanium Dental Implant with Machined Surface: An In Vitro and In Vivo Study

**DOI:** 10.3390/ma12132078

**Published:** 2019-06-28

**Authors:** Jun-Beom Lee, Ye-Hyeon Jo, Jung-Yoo Choi, Yang-Jo Seol, Yong-Moo Lee, Young Ku, In-Chul Rhyu, In-Sung Luke Yeo

**Affiliations:** 1 Department of Periodontology, Seoul National University School of Dentistry, Seoul 03080, Korea; 2 Department of Prosthodontics, School of Dentistry and Dental Research Institute, Seoul National University, Seoul 03080, Korea; 3 Dental Research Institute, Seoul National University, Seoul 03080, Korea

**Keywords:** dental implants, titanium, osseointegration, photofunctionalization, ultraviolet light, surface treatment

## Abstract

Ultraviolet (UV) photofunctionalization has been suggested as an effective method to enhance the osseointegration of titanium surface. In this study, machined surface treated with UV light (M + UV) was compared to sandblasted, large-grit, acid-etched (SLA) surface through in vitro and in vivo studies. Groups of titanium specimens were defined as machined (M), SLA, and M + UV for the disc type, and M + UV and SLA for the implant. The discs and implants were assessed using scanning electron microscopy, confocal laser scanning microscopy, electron spectroscopy for chemical analysis, and the contact angle. Additionally, we evaluated the cell attachment, proliferation assay, and real-time polymerase chain reaction for the MC3T3-E1 cells. In a rabbit tibia model, the implants were examined to evaluate the bone-to-implant contact ratio and the bone area. In the M + UV group, we observed the lower amount of carbon, a 0°-degree contact angle, and enhanced osteogenic cell activities (*p* < 0.05). The histomorphometric analysis showed that a higher bone-to-implant contact ratio was found in the M + UV implant at 10 days (*p* < 0.05). In conclusion, the UV photofunctionalization of a Ti dental implant with M surface attained earlier osseointegration than SLA.

## 1. Introduction

Dental implant restorations to replace missing teeth have become a routine practice in dental clinics. Using the implants as a prosthesis helps patients feel more comfortable and these implants are more functional compared to the traditional removable dentures [1,2,3]. For successful implant restorations, osseointegration must be achieved between the bone and the implant. Osseointegration is the direct structural and functional connection between living bone and the surface of a load-carrying implant [4], and it is an essential factor in achieving a successful implant. Generally, it is necessary to wait for several months after implant placement for osseointegration to be achieved [5]. Unsuccessful osseointegration leads to the early failure of implants, meaning that the implants cannot endure masticatory forces, resulting in implant mobility or pain [6,7]. This includes other time-consuming situations, such as an edentulous area with limited bone quantity, or problems in patients with osteoporosis, diabetes, cancer, irradiation, old age, and heavy smokers [8,9,10].

The original implant surface was a smooth machined surface, with an approximate Sa value of 0.5 μm [11,12]. This machined surface has certain advantages, including a simple manufacturing process (turning and polishing) and the ability to maintain a good hygienic state, resulting in a low incidence of peri-implant disease [12,13]. However, implants with a machined surface have shown a low bone-to-implant contact ratio (BIC) and a frequent failure of osseointegration before loading [14]. To enhance the osseointegration process, various surface modifying techniques have since been developed, where a roughened surface has demonstrated the best clinical long-term results. There are various roughening techniques, although the sandblasted, large-grit, acid-etched (SLA) surface is the most widely used and reported technique. The SLA surface sufficiently differentiates the pre-osteoblastic cells, enhances the osseointegration process, and leads to a higher BIC compared to the machined surface [15,16]. However, the roughened surface has been reported to accelerate plaque accumulation, wherein it is more difficult to remove plaque on the roughened surface than on the machined surface [12]. In this regard, reports show a greater incidence of peri-implant disease stemming from the use of the roughened surface compared to the machined surface [17].

Meanwhile, the photofunctionalization of implants using ultraviolet (UV) light has been highlighted as a simple and effective method to stimulate osseointegration in machined surfaces [18,19,20]. UV photofunctionalization is a phenomenon of changes occurring in titanium (Ti) surfaces after UV treatment. The process was discovered in 1977, where UV treatment transforms the natural hydrophobic properties of Ti surfaces into superhydrophilic properties by altering the physicochemical properties of the Ti. The process has been applied in environmental engineering and microbiology [21,22]. UV treatment creates the hydrophilic phase on the surface structure, thereby transforming the surface into a hydrophilic surface [23]. Photofunctionalization is also reported to enhance biological capabilities [18,19]. Consequently, the purpose of this study was to evaluate the effect of UV photofunctionalization on implants with a machined surface compared to the SLA surface, using an in vitro and in vivo study.

## 2. Materials and Methods

### 2.1. Ti Samples, Surface Analysis, and UV Treatment

#### 2.1.1. Preparation of the Ti Disc and Implant

In this experiment, commercially pure grade 4 Ti was tested in the shape of a disc (10 mm in diameter and 1 mm in thickness) and a screw-shaped implant (3.3 mm in diameter and 7 mm in length). The surface of the specimen was treated using the following methods: (a) M: The machined surface was turned and polished using sandpaper (600–1000 times); (b) SLA: The surface was sandblasted with alumina (Al_2_O_3_) particles, which were 50 μm in size and acid-etched, using hydrochloric acid and sulfuric acids (SLA surface; Point Implant Co., Seoul, Korea); and (c) M + UV: Machined surface treated with ultraviolet (UV) light. For the disc type, all the three surface treatments were examined for a negative control (M), a positive control (SLA), and an experimental group (M + UV). For the implant type sample, two surface treatments, i.e., the control (SLA) and the experimental group (M + UV), were investigated.

#### 2.1.2. Surface Analysis

Three samples were used in each group for each examination. We performed field emission scanning electron microscopy (FE–SEM; Hitachi S-4700, Hitachi, Tokyo, Japan) for a qualitative evaluation of the overall surface image. This was followed by a confocal laser scanning microscope (CLSM; LSM 800, Carl Zeiss AG, Oberkochen, Germany), where the surface roughness was quantitatively measured. The surface roughness parameters—arithmetical mean height (Sa), root mean square height (Sq), and developed interfacial area ratio (Sdr)—were measured at three randomly selected points in each sample. In addition, the chemical composition was analyzed using electron spectroscopy for chemical analysis (Sigma Probe, Thermo VG, East Grinstead, UK). Furthermore, the surface wettability of the Ti discs was examined using the contact angle from the sessile drop method, as measured by a contact angle meter (Pheonix 150, SEO, Kyunggido, Korea). All the procedures were performed under controlled conditions of 20 °C temperature and 46% humidity.

#### 2.1.3. UV Light Treatment

UV light treatment was achieved by irradiating the Ti discs in a specially manufactured generator using four 15 W bactericidal lamps (G15T8, Sankyo Denki, Tokyo, Japan), for at least 48 h. The intensity was approximately 5 mW/cm^2^ (λ = 254 ± 20 nm).

### 2.2. In Vitro Experiment

#### 2.2.1. Cell Culture

Murine pre-osteoblast MC3T3-E1 cells were purchased from ATCC (American Type Culture Collection; Manassas, VA, USA). The cells were seeded onto the discs (1 × 10^4^ cells/well) in a 12-well culture plate (Nunc, Roskilde, Denmark), and then cultured in α-minimum essential medium (α-MEM; Thermo Fisher Scientific, Waltham, MA, USA) supplemented with a 10% fetal bovine serum (FBS) and 1% penicillin/streptomycin. The cells were incubated at 37 °C under a humidified atmosphere of 95% air and 5% CO_2_. The culture medium was replaced every three days, and the osteogenic medium contained 10 mM β-glycerophosphate and 50 µg/mL ascorbic acid in the α-MEM.

#### 2.2.2. Cell Attachment

At 24 h after being seeded, the cell attachment was dual-stained using fluorescent dyes: 4′,6-diamidino-2-phenylindole (DAPI; Invitrogen, Carlsbad, CA, USA) and Alexa Fluor 568 phalloidin (Invitrogen, Carlsbad, CA, USA) to detect the nucleus and actin filaments, respectively. Fluorescence was visualized by a CLSM (LSM 800, Carl Zeiss AG, Oberkochen, Germany), and analyzed with the ZEN2010 software (Carl Zeiss, Oberkochen, Germany).

#### 2.2.3. Cell Proliferation

The proliferative activity of cells was measured using a methyl thiazolyl tetrazolium (MTT) assay (Sigma-Aldrich, St. Louis, MO, USA) at 1, 3, and 7 days after being seeded. The culture media was replaced with an MTT solution and incubated for 3 h at 37 °C. After removing the MTT solution, 0.5 mL of 10% dimethyl sulfoxide in isopropanol (iDMSO) was added for 30 min at 37 °C. Then, the proliferation rate was assessed by its optical density (OD) at 570 nm. The value of the OD was measured using a microplate reader (BioTek, Winooski, VT, USA).

#### 2.2.4. Cell Differentiation

Total RNA in the cell cultures was extracted using the TRIzol method described by Chomczynski at 1, 4, 7, 10, and 14 days after osteoblast differentiation [24]. A reverse transcriptase–polymerase chain reaction (RT–PCR) was performed with primer sets for type I collagen (Col), alkaline phosphatase (Alp), and osteocalcin (Ocn), as described in Table 1. Quantitative real-time PCR was performed using a Takara SYBR premix Ex Taq (Takara Bio, Kusatsu, Japan) on a 7500 real-time PCR system (Applied Biosystems, Foster City, CA, USA). Each primer contained a final concentration of 200 nM, and a quantity of cDNA corresponding to 50 ng of total RNA. The PCR primers were synthesized using Integrated DNA Technology (Coralville, IA, USA). According to the manufacturer’s instructions, the PCR cycling conditions comprised 40 cycles at 95 °C for 5 s, and 60 °C for 34 s after denaturation at 95 °C for 30 s. The cycle threshold (Ct) values were acquired using the automated threshold analysis in the Sequence Detection software version 1.4 (Applied Biosystems, Foster, CA USA). Each target mRNA expression was calculated using the comparative cycle threshold method according to the manufacturer’s instructions. The relative mRNA expression levels were normalized to glyceraldehyde-3-phosphate dehydrogenase (GAPDH). The GAPDH mRNA expression levels remained steady during the osteoblast differentiation, showing similar Ct values.

### 2.3. In Vivo Experiment

#### 2.3.1. Animals

The rabbit tibia model was used. All the procedures were conducted with the approval of the Ethics Committee of Animal Experimentation of the Institutional Animal Care and Use Committee (CRONEX-IACUC 201803003; Cronex, Hwasung, Korea), according to the guidelines of Animal Research: Reporting In Vivo Experiments (ARRIVE).

Thereafter, four female New Zealand white rabbits (3–4 months old and 2.5–3.0 kg in weight) were anesthetized via a 1 mL intramuscular injection with a dose of 15 mg/kg of tiletamine/zolazepam (Zoletil 50, Vibrac Korea, Seoul, Korea) and 5 mg/kg of xylazine (Rompun, Bayer Korea, Seoul, Korea). Then the tibiae of the rabbits were shaved and disinfected with povidone iodine solution. Local anesthesia was administered in the surgical area with 2% lidocaine containing 1:100,000 epinephrine (Yuhan Co., Seoul, Korea).

#### 2.3.2. Surgical Procedure

A full-thickness flap was made on the medial side of both tibiae, followed by exposure of the underlying bone. In each tibia, two holes for implant placement were drilled bicortically using implant surgical drills under copious sterile saline irrigation. The diameter of the drills was increased sequentially, with a final drill diameter of 2.8 mm. Then, the implant with a diameter of 3.3 mm was inserted into the hole and engaged bicortically with sufficient stability. The SLA and M + UV implants were allocated to each hole based on a 2 × 2 Latin square randomization. Following the implant placement, the periosteum and fascia were sutured with 4-0 resorbable polyglactin material (Vicryl, Ethicon, Somerville, MA, USA), and the skin was sutured with 4-0 monofilament nylon (Blue nylon, Ailee, Busan, Korea). Post-operatively, each rabbit was kept in a separate cage and administered with 5 mg/kg of enrofloxacin (Komibiotril, Komipharm International Co., Siheung, Korea) for seven days.

#### 2.3.3. Sacrifice and Microcomputed Tomography (Micro-CT)

**Figure 1:**
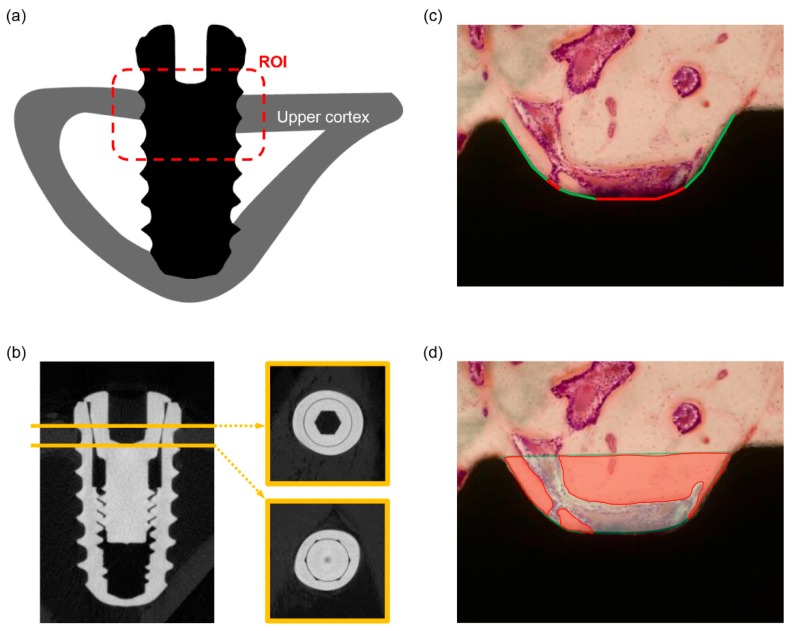
(**a**) The schematic drawing of the region-of-interest (ROI). The ROI was defined as an area within threads engaged in the upper cortical bone (red dot box); (**b**) microcomputed tomography (micro-CT) images for the measurement of the bone-to-implant contact ratio (BIC); (**c**) the bone-to-implant contact ratio (BIC) was calculated by the length of the green line divided by the total length of the well (green and red line); (**d**) the definition of the bone area (BA) was calculated by the area of red color divided by the total area of the well.

Two experimental animals were sacrificed at 10 days and the other two animals at 28 days after the surgery by an intravenous overdose of potassium chloride. The implants were surgically harvested en bloc with the surrounding bone. Then the implant–bone blocks were immediately immersed in a 10% neutral buffered formalin fixative. Micro-CT imaging was performed using a SkyScan 1275 (Bruker microCT, Kontich, Belgium). The X-ray source was set at an acceleration voltage of 100 kV and a pixel size of 10 μm. Each sample was scanned three times, using 360° spiral scanning on the SkyScan 1275 with a scanning time of 2 h. Reconstruction was performed using an NRecon (v. 1.7.3.2, Bruker microCT). The region-of-interest (ROI) was defined as the area within consecutive threads engaged in the upper cortical bone (Figure 1a). The analysis was performed using the CTAn software (v. 1.18.4.0, Bruker microCT; Figure 1b), and it also involved the visualization software DataViewer (v. 1.5.4.0, Bruker microCT) and CTVox (v. 3.3.0, Bruker microCT).

#### 2.3.4. Histological Preparation and Histomorphometric Measurement

After μCT scanning, the implant–bone blocks were dehydrated in a series of ethanol with increasing concentrations and then embedded in light-curing resin (Technovit 7200 VLC, Hereaus Kulzer, Hanau, Germany). The embedded blocks were sectioned perpendicular to the longitudinal axis of the implant using the EXAKT system (EXAKT Apparatebau, Norderstedt, Germany), following the method described by Donath and Breuner [25]. The section was then ground to a thickness of 40 μm and stained with hematoxylin and eosin (H&E) for examination using a light microscope. These undecalcified, ground sections were observed under a light microscope (BX51, Olympus, Tokyo, Japan) to measure the BIC and bone area (BA; Figure 1c,d). The region-of-interest (ROI) was defined as the same area that was used in the micro-CT analysis. The measurement was performed under a ×100 magnification, using the SPOT software version 4.0 (Diagnostic Instruments, Sterling Heights, MI, USA) and Image-Pro Plus (Media Cybernetics, Rockville, MD, USA).

### 2.4. Statistical Analysis

The Kruskal–Wallis test was used to evaluate statistically significant differences amongst the three groups of discs. If there was a significant difference amongst the three groups, the post-hoc Tukey method was applied. To compare the two groups of implants, the Mann–Whitney U test was performed to determine the statistically significant differences. *p* < 0.05 was set as the statistical significance. All statistical analyses were performed using SPSS 20.0 (IBM Corp., Armonk, NY, USA).

## 3. Results

### 3.1. Surface Characteristics

**Figure 2:**
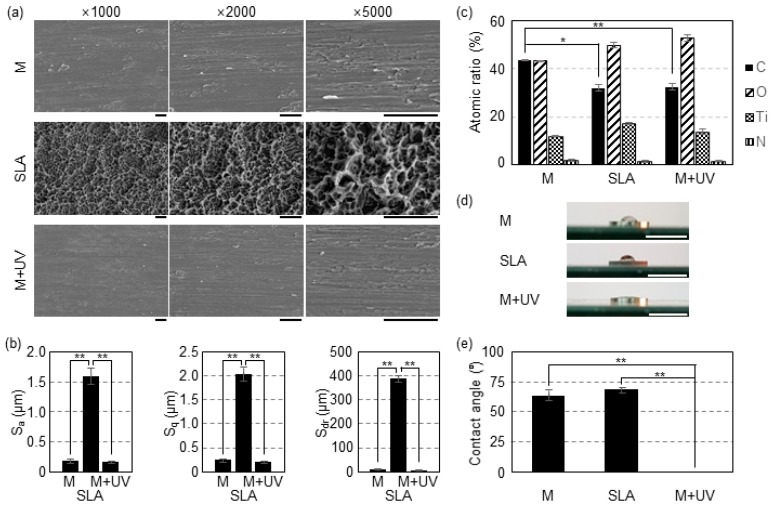
(**a**) Scanning electron microscopy (SEM) images of the Ti discs. M: Machined surface (top row); SLA: Sandblasted with large grit and acid etched surface (middle row); M + UV: machined surface treated with ultraviolet light (bottom row). Scale bars: 10 μm. (**b**) Surface roughness (Sa and Sq) and surface area ratio (Sdr) of the Ti discs evaluated by confocal laser scanning microscopy (CLSM) analysis. The values for the M and M + UV surfaces are similar and smaller than the SLA. (**c**) Element content of the surfaces of the Ti disc according to the energy dispersive x-ray spectrometer (XPS). M + UV shows a significantly lower carbon percentage than the M disc, but there is no significant difference between the SLA and M + UV. (**d**) The changes in wettability of the Ti discs. Superhydrophilicity after UV treatment for 48 h was observed. Scale bars: 10 mm. (**e**) The value of the contact angle of the Ti discs. Without UV treatment, the Ti disc was hydrophobic, but became superhydrophilic (zero degree angle) after UV treatment for 48 h. Error bars show the standard deviation. (*) and (**) represents significance compared with each pair, *p* < 0.05 and *p* < 0.01, respectively.

**Figure 3:**
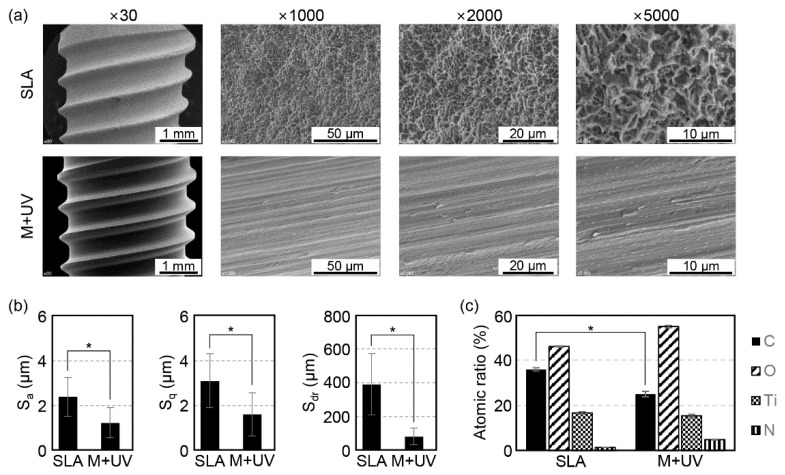
(**a**) Scanning electron microscopy (SEM) images of the Ti implants. SLA: Sandblasted with large grit and acid etched surface (top row); M + UV: Machined surface treated with ultraviolet light (bottom row). Magnification: ×30, ×1000, ×2000, and ×5000 from the left. (**b**) Surface roughness (Sa and Sq) and surface area ratio (Sdr) of the Ti discs according to the confocal laser scanning microscopy (CLSM) analysis. The values of the M + UV surfaces are smaller than the SLA, which means it is smoother than the SLA. (**c**) Element content of the surfaces of the Ti discs according to the energy dispersive x-ray spectrometer (XPS). The M + UV discs contain half of the carbon percentage of the SLA discs. Error bars show the standard deviation. (*) represents the significance compared with each pair, *p* < 0.05.

In the overall evaluation of the Ti samples via the FE–SEM images, the M and M + UV surfaces showed similar evidence of machine turning (continuous straight traces) with smooth surfaces, although the SLA surface presented a rougher surface with a typical honeycomb appearance (Figure 2a and Figure 3a).

Surface roughness parameters for the samples are shown in Figure 2b and Figure 3b. In the disc specimen, the SLA surface showed higher Sa, Sq, and Sdr values, and these were significantly different from the M and M + UV surfaces (*p* < 0.01). There was no statistical difference between the M and M + UV surfaces (*p* > 0.05). Similarly, for the implant specimen, the M + UV and SLA surfaces were statistically different across all the parameters (*p* < 0.05).

Chemical compositions from the x-ray spectrometer (XPS) revealed that, compared with the 43.42% ± 0.31% carbon in the M disc, the M + UV disc contained about 32.35% ± 1.50% carbon, which was statistically different (*p* = 0.000). The SLA showed 31.87% ± 1.42% carbon, with no significant difference from the M + UV disc (*p* = 0.051). On the other hand, the M + UV implant showed a significantly lower carbon percentage compared to the SLA implant (*p* = 0.049; Figure 2c and Figure 3c).

Contact angle measurement was performed for the disc-type specimens. The M and SLA discs showed a hydrophobic status with angles of 63.6 ± 4.7° and 68.3 ± 2.5°, respectively. On the other hand, 48 h after UV treatment, the M + UV discs showed superhydrophilicity at a 0° contact angle (*p* = 0.000; Figure 2d,e).

### 3.2. In Vitro Test

#### 3.2.1. Cell Attachment

**Figure 4:**
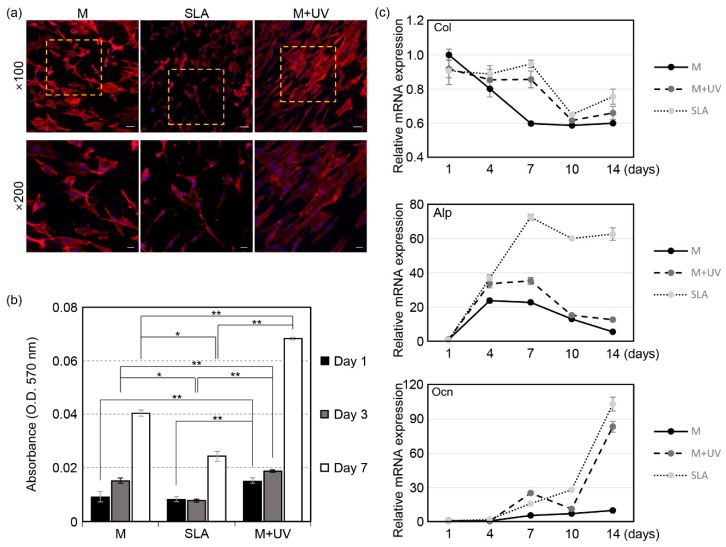
(**a**) Confocal microscopic images of the MC3T3-E1 cells, 24 h after being seeded on the Ti discs. The areas in the dotted box are magnified in the bottom row. Scale bars: 50 μm at ×100 and 20 μm at ×200 magnification. (**b**) Evaluation of cell proliferation of the MC3T3-E1 cells by an MTT assay at 1, 3, and 7 days after being seeded on the Ti discs. (**c**) Evaluation of the cell differentiation of MC3T3-E1 cells by real-time PCR at 1, 4, 7, 10, and 14 days after being seeded on the Ti discs. The relative mRNA expression levels were normalized to glyceraldehyde-3-phosphate dehydrogenase (GAPDH). The osteogenic markers are type I collagen (top), alkaline phosphatase (ALP, middle), and osteocalcin (OCN, bottom). UV photofunctionalization enhanced the osteoblastic gene expression. Error bars show the standard deviation. (*) and (**) represent the significance compared with each pair, *p* < 0.05 and *p* < 0.01, respectively.

The CLSM images of the cells are shown in Figure 4a. A wider spread of cells was observed in the M + UV groups compared to the other groups. In the SLA discs, the cells were sharp and needle-like shaped, implying that the osteoblasts were not prone to attach to the SLA surfaces.

#### 3.2.2. Cell Proliferation

The MTT assay showed that the amount of cells increased in a time-dependent way on all the surfaces. On the M + UV surface, the cells proliferated more significantly than the other surfaces at days 1, 3, and 7, with a *p*-value of less than 0.01, as shown in Figure 4b. On day 7, the amount of cells on the M + UV surface was two times greater than cells on the SLA surface (0.068 ± 0.0005 vs. 0.040 ± 0.001, *p* = 0.000). In particular, at day 3 and 7, the cells proliferated more on the M surface than on the SLA surface.

#### 3.2.3. Quantitative Assessment of the Osteogenic Markers

Figure 4c shows the relative mRNA expression of Col, Alp, and Ocn. The RT–PCR analysis showed that Col was more significantly expressed on the M + UV and SLA surfaces at days 7, 10, and 14 compared to the M surface, although the M + UV and SLA surfaces were not significantly different. The expression level of Alp on the SLA surface was not different from the M + UV surface at days 1 and 4, but it was significantly higher than the expression levels at days 7, 10, and 14. The M + UV surface expressed the Alp gene more than the M surface at days 4, 7, and 14. The expression level of Ocn on the M + UV surface was significantly higher at day 7, but it was lower at days 10 and 14.

### 3.3. In Vivo Test

#### 3.3.1. Histomorphometry

**Figure 5:**
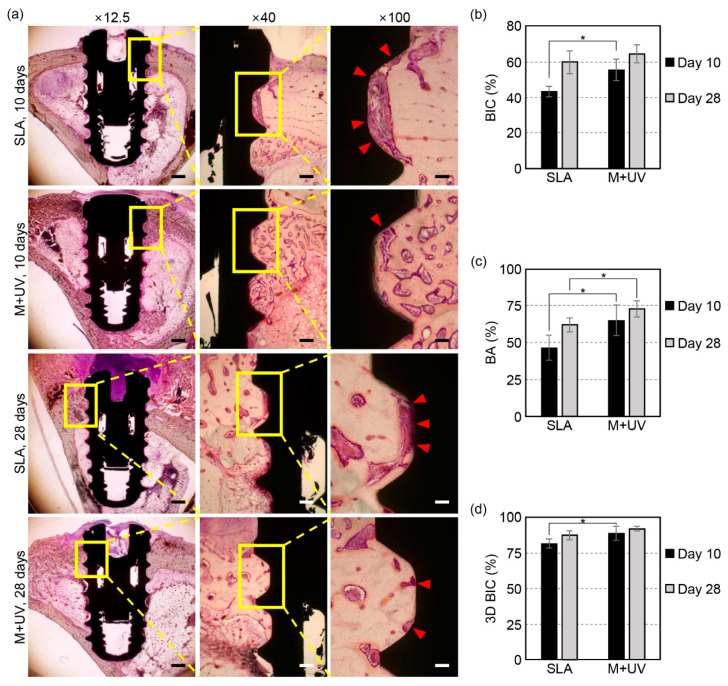
(**a**) Representative histologic sections of the rabbit tibia at 10 and 28 days after the implant placement. In the SLA implant, the osteoblast and organic matrix, which had not mineralized yet, was more observable on the interface between the bone and implant compared to the M + UV implant (red arrow head; magnification ×12.5, ×40, and ×100 from the left, hematoxylin and eosin staining). The scale bars: 1 mm at ×12.5, 200 μm at ×40, and 100 μm at ×100 magnification. (**b**) The bone-to-implant contact ratio (BIC) was evaluated histologically at days 10 and 28. The M + UV implant shows a significantly higher BIC than the SLA at day 10, but there is no significant difference at day 28. (**c**) The bone area ratio (BA) evaluated histologically at 10 and 28 days. The M + UV implants show significantly more BA than the SLA at days 10 and 28. (**d**) The bone-to-implant contact ratio evaluated by micro-CT (3D BIC) at days 10 and 28. The M + UV implants show a significantly higher 3D BIC than the SLA at day 10, but there is no significant difference at day 28. Error bars show the standard deviation. (*) represents the significance compared with each pair, *p* < 0.05.

All the implants were successfully osseointegrated at days 10 and 28 (Figure 5a). At day 10, the BIC ratios of the M + UV implants (55.93% ± 6.19%) were significantly higher than that of the SLA implants (43.38% ± 3.20%, *p* = 0.021). However, at day 28, the BIC ratios of the M + UV implants (64.88% ± 5.35%) were not significantly different from that of the SLA implants (59.93% ± 6.44%, *p* = 0.149; Figure 5b).

In terms of the BA, the M + UV implants were significantly higher compared to the SLA implants (46.55% ± 8.59%) at day 10 (65.09% ± 10.42% vs. 46.55% ± 8.59%, *p* = 0.042) and at day 28 (72.70% ± 5.52% vs. 61.83% ± 4.89%, *p* = 0.043; Figure 5c).

#### 3.3.2. Micro-CT

The three-dimensional BIC was evaluated using micro-CT. The micro-CT analysis revealed that the three-dimensional BIC of the M + UV implants was significantly higher than that of the SLA implants at day 10 (88.87% ± 5.1% vs. 81.6% ± 3.28%, *p* = 0.046), but it was not statistically different at day 28 (91.91% ± 1.55% vs. 87.47% ± 2.93%, *p* = 0.201; Figure 5d).

## 4. Discussion

In this study, we found that UV photofunctionalization on a Ti screw-shaped implant with an M + UV surface showed a higher BIC than the SLA surface at day 10, and there was no significant difference at day 28. This was confirmed in both two-dimensional and three-dimensional measurements. The results indicated that the UV photofunctionalization could accelerate the osseointegration process, and achieve firm fixation between the implant and the surrounding bone earlier. These findings are supported by other studies, where Park et al. found that after four months of healing, the UV-treated implants in rabbits showed a higher BIC than the untreated implants. The authors observed that UV treatment decreased both carbon impurities on the surface and water contact angles [26]. Similarly, Aita et al. showed that the UV-treated acid-etched implants at week two had a push-in value equivalent to the untreated acid-etched implant [18]. Pyo et al. measured the removal torque test in UV-treated implants and showed that it was 50% higher than in untreated implants [27]. Hirota et al. retrospectively studied and found that the use of photofunctionalization reduced the risk of early implant failure with an odds ratio of 0.30 (*p* < 0.05) [28]. Soltanzadeh studied the effect of UV photofunctionalization on immediately loaded implants in a rat model. After the placement, the implants were immediately loaded with 0.46 N of static lateral force. The results showed that osseointegration was successful in 100% of photofunctionalized implants, but 28.6% of untreated ones. The value of the push-in test was 2.4 times higher in photofunctionalized implants [29].

Histologically, the BA was significantly higher at days 10 and 28 in the M + UV compared to the SLA implants, meaning that the M + UV implant had a higher amount of mineralized bones between threads of implants. Pyo et al. evaluated the osteogenic dynamics using fluorescent labeling at four weeks after implant placement; and found that in the UV-treated implant, the interfacial areas between the bone and implant and the areas within the threads were filled with calcein-positive tissues compared to the untreated implants. This meant that UV photofunctionalization could lead to earlier bone deposition [27]. Ueno et al. showed that the UV-treated acid-etched implant had a marked bone formation in a gap healing model without cortical support [20]. Kitajima et al. measured the implant stability quotients (ISQ) for 55 photofunctionalized implants with low and extremely low initial stability at the time of placement and stage-two surgery. Then they calculated the ISQ increase per month, defining the osseointegration speed index (OSI). The OSI ranged 3.9–4.7 substantially higher than the OSIs for untreated implants reported in other literatures (0.36–2.8) [30]. Ijishima et al. evaluated the effect of photofunctionalization on aged rats. The aged rats showed considerably lower biological capabilities (cell attachment, proliferation, and ALP activity) than the young. However, the enhancement of cell attachment and differentiation were observed on the photofunctionalized Ti discs compared with untreated one. Moreover, in the femurs of aged rats, the photofunctionalized mini-implant showed the higher push-in value than untreated one after two weeks of healing. These findings supported that UV photofunctionalization could be also valuable in the compromised sites [31].

Generally, the surface roughness is considered as a main factor for the improvement of osseointegration. However, in this study, UV treatment did not physically change any surface roughness as shown in the SEM and CLSM. Rather, it induced superhydrophilicity (0° angle), reduced the percentage of hydrocarbons, and increased the osteoblast proliferation, attachment, and differentiation, as shown in the in vitro study. This indicated that the only physico-chemical changes in the Ti surface could enhance the biological activities. In the XPS analysis, Roy et al. found that UVC photon energy decreased carbon deposition and the amount of H_2_O on Ti surface, and produced many –OH groups (TiOH) without any changes in surface topography. They explained that, through these chemical changes, the UV photofunctionalization could create the superhydrophilicity of Ti. The improvement of biological capabilities by UV photofunctionalization was supported by other studies [32]. Aita et al. showed that Col and osteopontin (Opn) were more expressed in the UV-treated discs [18]. The RT–PCR analysis performed by Zhang et al. showed that the expression of genes encoding Col, Runx2, BMP, and Opn increased in the UV-treated surface [33]. In contrast, Att et al. assessed the RT–PCR of genes for Opn and Ocn in bone marrow cells derived from the femur of eight-week-old male Sprague-Dawley rats, and found that there was no significant difference at days 10 and 20. The differences may have been caused by the kinds of cells, time points, and intensity and wavelength of the UV generator. Further research is required to elucidate this aspect.

With regard to plaque accumulation and peri-implant disease, the implant with a smooth surface is considered to be superior to an implant with a rough surface. Berglundh et al. observed that, at five months after the removal of ligature, bone loss accelerated in the SLA implant but not in the polished implant. Histologically, the size of the inflammatory lesion and the area of plaque were larger in the SLA surface [12]. Additionally, Albouy et al. compared the turned and the roughened implant (Ti-Unite), at six months after the ligature removal, and observed a larger amount of bone loss in the Ti-Unite implant compared to the turned implant (1.47 mm vs. 0.3 mm). This meant that spontaneous progression of peri-implantitis had occurred in the implant with a rough surface [17]. However, the machined implant had a definite drawback in that it had a low BIC level, because the osteoblastic differentiation was lower compared to the smooth surface [34,35]. Therefore, the enhancement of osteoblastic differentiation on the Ti with an M surface by UV photofunctionalization is considered to be inspired. Additionally, UV photofunctionalization itself could decrease plaque formation on Ti surface. De Avila et al. found that after 16 h incubation, there were significantly lower oral bacterial attachment on the UV-treated Ti disc compared to the untreated one [36].

The hydrophilicity of the implant surface can be induced by UV photofunctionalization [18,19,20,27,37] or preservation in a storage medium [38,39,40]. Both methods are effective and can improve the bone healing process and attain early osseointegration. However, the latter method has been reported to lead to foreign deposition and little elimination of hydrocarbons on the Ti surface. Moreover, the saline storage method is inferior to UV photofunctionalization in osteoblast spreading and adhesion [41]. Considering this point, UV treatment is considered a safer method to modify the implant surface to make it hydrophilic. On the other hand, Att et al. mentioned that, in UV photofunctionalization, the superhydrophilicity is not a significant factor in explaining the higher BIC in the UV-treated Ti discs compared to the acid-etched ones. The elimination of hydrocarbon on the surface was considered to be a significant factor [19]. The aging of the Ti is related to the contamination and accumulation of the hydrocarbon on the Ti surface, and it can suppress cell recruitment and biologic activity [42,43].

The combination of variables such as duration, intensity, and wavelength can create various modes of UV photofunctionalization, noting that the optimal combination is a controversial issue. The exposure time has been used from 12 min to 48 h [44,45]. However, Aita et al. and Att et al. have shown that, between 24 and 48 h, there was an increase in hydrophilicity and biological effects [18,19]. Additionally, treatment with UVC (λ = 240 ± 40 nm) has shown more biological improvements compared to UVA (λ = 360 ± 40 nm). Consequently, in our experiment, to maximize the effects of UV, the mode of UV photofunctionalization was determined as UVC treatment for 48 h.

There are still several questions regarding UV photofunctionalization. Strictly, the UV light treatment used in this study may be called physical photo-activation, rather than functionalization, because no chemical application to the surface, which have been shown in the previous studies, were used for enhanced bone response [11,46]. More obvious concept of the UV surface treatment needs to be established in the physical and chemical aspects. Amongst several factors following UV photofunctionalization, we also still need to identify the main factors for the enhancement of biological activity, superhydrophilicity, and removal of hydrocarbon. If they contribute to the improvement, there is a need to understand the mechanism through which they are inter-connected. Therefore, further studies are needed to fully appreciate the effects of UV photofunctionalization.

## 5. Conclusions

Within the limitations of the present study, UV photofunctionalization of a Ti dental implant with an M surface attained an earlier osseointegration compared to an implant with an SLA surface. The enhancement was considered to result from the superhydrophilicity, the elimination of hydrocarbon on the surface, and the improvement of osteoblastic activities.

## Figures and Tables

**Table 1:** Primer sequences for the reverse transcriptase–polymerase chain reaction (RT–PCR).

Gene	Forward Primer (5′-3′)	Reverse Primer (5′-3′)
Col ^1^	GCTCCTCTTAGGGGCCACT	CCACGTCTCACCATTGGGG
Alp ^2^	GGCTACATTGGTCTTGAGCTTTT	CCAACTCTTTTGTGCCAGAGA
Ocn ^3^	CTGACAAAGCCTTCATGTCCAA	GCGCCGGAGTCTGTTCACTA

^1^ Type I collagen; ^2^ Alkaline phosphatase; ^3^ Osteocalcin.
